# Mapping the Outcomes of Low-Vision Rehabilitation: A Scoping Review of Interventions, Challenges, and Research Gaps

**DOI:** 10.3390/vision10010003

**Published:** 2026-01-12

**Authors:** Kingsley Ekemiri, Onohomo Adebo, Chioma Ekemiri, Samuel Osuji, Maureen Amobi, Linda Ekwe, Kathy-Ann Lootawan, Carlene Oneka Williams, Esther Daniel

**Affiliations:** 1 Department of Optometry and Vision Sciences, Abia State University, P.M.B. 2000, Uturu 440001, Abia State, Nigeria; drsamsuji@yahoo.com (S.O.); mauryn55@gmail.com (M.A.); lindaekwe386@gmail.com (L.E.); 2 Department of Optometry and Vision Sciences, The University of the West Indies St. Augustine, West Indies, St. Augustine P.O. Box 999183, Trinidad and Tobago; 3 Department of Optometry and Vision Sciences, University of Benin, P.M.B. 1154, Benin 300001, Edo State, Nigeria; onohomo@yahoo.com; 4 Department of Optometry and Vision Science, University of Guyana, P.O. Box 101110, Georgetown 4130106, Guyana; 5 Department of Optometry, Imo State University, P.M.B. 2000, Owerri 460231, Imo State, Nigeria; 6 School of Nursing, Faculty of Medical Sciences, The University of the West Indies St. Augustine, West Indies, St. Augustine P.O. Box 999183, Trinidad and Tobago; kathy-ann.alphonso-lootawan@uwi.edu (K.-A.L.); esther.daniel@uwi.edu (E.D.); 7 Department of Public Health, Federal University of Technology, P.M.B. 1526, Owerri 460231, Imo State, Nigeria; 8 Department of Nursing, North Central Regional Health Authority, P.O. Box 350, Champs Fleurs 9807,Trinidad and Tobago; miss_carlene@hotmail.com

**Keywords:** low vision, vision rehabilitation, intervention outcomes, quality of life, scoping review

## Abstract

**Introduction:** Low vision affects more than visual acuity; it substantially disrupts daily functioning and may contribute to long-term cognitive, emotional, and social consequences. When medical or surgical treatment options are no longer effective, structured low-vision rehabilitation becomes essential, providing strategies and tools that support functional adaptation and promote independence. This review aims to map the current outcomes of rehabilitation services, identify gaps in existing research, and highlight opportunities for further study. **Methods:** An article search was conducted via PubMed, Scopus, PsycInfo, and Google Scholar. Then, title, abstract, and full-text screenings for inclusion were performed by all the authors independently, and disagreements were resolved through discussion. The relevant outcomes from the eligible publications were extracted by four authors and then cross-checked by the other authors. The results are presented via the Preferred Reporting Items for Systematic Reviews and Meta-analysis extension for Scoping Reviews checklist. **Results:** A total of 13 studies met the inclusion criteria. Most were randomized controlled trials (*n* = 10,77%), with the majority conducted in the United States and the United Kingdom. Study populations consisted of adults aged 18 years and older. Across the included studies, low-vision rehabilitation interventions particularly visual training, magnification-based programs, and multidisciplinary approaches, were associated with significant improvements in visual function, activities of daily living, and vision-related quality of life. **Conclusions:** Low vision rehabilitation interventions demonstrate clear benefits for visual acuity, contrast sensitivity, reading speed, and functional independence. However, substantial gaps remain, including limited evidence on long-term outcomes, inconsistent assessment of psychosocial influences, and underrepresentation of diverse populations. Standardized outcome measures and long-term, inclusive research designs are needed to better understand the sustained and equitable impact of low-vision rehabilitation.

## 1. Background

Low vision is defined as a significant vision impairment that cannot be fully corrected using standard eyeglasses or through medical or surgical interventions. This condition can result from various ocular diseases, such as glaucoma, macular degeneration, or diabetic retinopathy, as well as neurological disorders, including stroke or traumatic brain injuries [1]. Globally, low vision is a major public health concern. It is estimated that approximately 285 million people are visually impaired [2], highlighting the scale of this issue. The effects of low vision extend beyond the inability to see well; they pose significant challenges in daily life. Individuals with low vision often struggle with reading, which can hinder their ability to engage with information, culture, and educational resources. Furthermore, limitations in physical activities often lead to social participation restrictions, making it difficult for them to engage with their communities and maintain social connections [3]. Beyond the immediate challenges, low vision can have serious long-term consequences. Research indicates a strong correlation between vision impairment and cognitive decline, including the risk of developing dementia. The reduction in quality of life is another critical factor for those living with low vision, as feelings of frustration, isolation, and dependency can significantly affect mental health and emotional well-being [4,5,6]

To comprehensively assess the impact of rehabilitation interventions, this study adopts the International Classification of Functioning, Disability, and Health (ICF) framework, which conceptualizes disability as an interaction between an individual’s health condition and contextual factors [7]. The ICF framework categorizes rehabilitation outcomes into (1) Body Functions and Structures (e.g., visual impairment severity), (2) Activity Limitations (e.g., reading difficulties, mobility issues), (3) Participation Restrictions (e.g., social isolation, employment challenges), and (4) Environmental and Personal Factors (e.g., assistive device accessibility, psychological adaptation). This framework provides a structured approach to understanding how low-vision rehabilitation interventions affect functional independence, social participation, and quality of life.

When conventional methods such as surgery or medication are no longer viable options for improving vision, alternative approaches can be implemented to assist individuals in adapting to their condition. Vision rehabilitation plays a pivotal role in this adjustment process. It encompasses a range of services designed to empower people with vision loss and help them maintain their in- dependence. Furthermore, these programs often involve family members, friends, and caregivers, equipping them with the knowledge and skills to provide support [7].

Vision rehabilitation includes training in the use of adaptive technologies, such as magnifiers or screen readers, as well as nonvisual techniques for accomplishing daily tasks. It may also involve orientation and mobility training, which helps individuals navigate their environment safely. In addition, counseling services are often part of rehabilitation programs, addressing emotional challenges and fostering coping strategies [3,4]. Despite the availability of rehabilitation services, low vision presents multifaceted challenges that necessitate a comprehensive approach to support those affected. A scoping review of these interventions is crucial to address the clinical, psychological, and social aspects of low vision through rehabilitation and supportive measures so that individuals can find ways to adapt and thrive despite their visual impairments. Such analysis will provide valuable insights for healthcare providers, policymakers, and researchers, helping to guide the rehabilitation service. This review aims to map the current outcomes of rehabilitation services, identify gaps in existing research, and highlight opportunities for further study.

## 2. Review Questions

1. What are the outcomes of low-vision rehabilitation interventions for adults?
2. What types of studies have been conducted to evaluate the outcomes of low-vision rehabilitation interventions for adults?
3. What are the existing research gaps in the field of low-vision rehabilitation interventions for adults that require further exploration?

## 3. Methods and Materials

This scoping review was guided by the framework presented by Arksey and O’Malley and used an iterative approach to include studies [8]. The framework starts by identifying the research question, selecting studies, and charting data.

## 4. Registration Statement

This review was prospectively registered on the Open Science Framework (OSF) under the title “Mapping the Outcomes of Low Vision Rehabilitation: A Scoping Review of Interventions, Challenges, and Research Gaps” (registration DOI: https://osf.io/nd2qy accessed on 4 November 2025). The registration details include the study rationale, objectives, and methodological approach, following the PRISMA-ScR guidelines. Any deviations from the original protocol have been transparently reported within this manuscript.

## 5. Eligibility Criteria

- Study period: This study included studies published up to December 2024.
- Study type: This study included a cross-sectional study, clinical trials, cohort studies, and a case report.
- Language: this study included studies published in the English language.
- Population: This study included studies that were conducted among adults.
- The study included both published and unpublished articles conducted globally.
- Publications without the full text, reviews, and meta-analyses were excluded.

## 6. Information Sources and the Search Strategy

A comprehensive search was conducted across PubMed, Scopus, Web of Science, and Google Scholar using the Cochrane acronym Population, Concept, and Context (PCC) as guidance for the retrieval process from these databases. Medical Subject Headings (MeSH) terms, along with Boolean operators such as “AND” and “OR”, were used. The search terms included the following: adult OR elderly OR old OR Low vision OR visual impairment OR vision loss OR Ophthalmic conditions OR Low vision status OR declining vision OR vision loss OR Visual field loss OR poor vision OR rehabilitation (Supplementary File S1). Additionally, manual searches were conducted of the references of the retrieved articles. The research and reporting methods of the scoping review were consistent with the preferred reporting items for systematic reviews and the meta-analysis extension for scoping reviews (PRISMA-ScR) (Supplementary File S2). The search terms were run in two electronic databases and were adapted to the required format from medical subject headings for the search in PubMed (MeSH Terms). The references that were retrieved through the online search were imported to EndNote and duplicates were removed. Additional relevant references were subsequently identified manually from the reference lists of the publications eligible for full-text screening.

## 7. Selection of the Sources of Evidence and Data Items

All publications were evaluated according to predefined eligibility criteria. The screening process was conducted independently by all reviewers in two stages: (1) title and abstract screening, followed by (2) full-text assessment. After each screening stage, the reviewers compared their selections and resolved differences through discussion. When discrepancies remained, a third reviewer was consulted to reach consensus. Title/abstract and full-text screening were therefore performed independently by all authors. Data extraction was carried out by four reviewers working independently, and the extracted information was subsequently cross-checked by the remaining authors to ensure completeness and accuracy.

## 8. Data Charting Process

In this study, the data extraction tool was developed by expanding the 22 items from the PRISMA-ScR checklist. These items included the author’s name, year of publication, study setting, study population, study design, outcomes, interventions, comparators, research gaps, and key findings. The extraction tool was refined following a pilot exercise and subsequently used to guide full-text data extraction for all eligible studies. Relevant outcomes were independently extracted by four authors and cross-checked by the remaining authors to ensure accuracy. For each included study, we documented whether outcomes were self-reported (e.g., QoL instruments) or clinician-measured (e.g., visual function tests, ADL assessments). Intervention characteristics were also systematically extracted, including the type of rehabilitation provided, delivery format, provider(s), setting, and specific techniques employed. 

## 9. Critical Appraisal of Included Studies

Although formal critical appraisal is not mandatory for scoping reviews, a descriptive methodological assessment was performed to enhance transparency and contextualize the interpretability of findings across the included studies.

### 9.1. Methodological Quality

The majority of the included studies were randomized controlled trials, but reporting quality varied. Several trials lacked detailed descriptions of randomization procedures, allocation concealment, or masking, introducing potential sources of bias. Cohort studies frequently relied on convenience samples, limiting generalizability.

### 9.2. Clarity of Intervention Reporting

Interventions were inconsistently described across studies. While some provided detailed protocols for visual training, home-based rehabilitation, or multidisciplinary programs, others reported only broad descriptions, making replication difficult. The level of therapist expertise and training duration were often unclear.

### 9.3. Appropriateness of Outcome Measures

Outcome measurement tools varied widely, including visual acuity charts, contrast sensitivity tests, reading speed assessments (MNREAD, IReST), quality-of-life instruments (VFQ-48, LVQOL), and activity-based measures (GAS, ADL scales). Although most measures were validated, inconsistent application across studies limited comparability.

### 9.4. Risk of Bias Indicators

Common risks of bias included:

- Allocation bias: Incomplete description of randomization in several RCTs.
- Performance bias: Blinding was rarely feasible due to the nature of rehabilitation interventions.
- Attrition bias: Some studies had notable dropout rates without sensitivity analyses.
- Detection bias: Outcome assessors were not always masked to treatment allocation

## 10. Synthesis and Analysis of the Results

To systematically categorize and interpret the outcomes of low-vision rehabilitation, this study applied the International Classification of Functioning, Disability, and Health (ICF) framework. The ICF framework conceptualizes rehabilitation outcomes across four domains: (1) Body Functions and Structures, which include the physiological impact of low vision; (2) Activity Limitations, referring to difficulties in performing daily tasks such as reading or mobility; (3) Participation Restrictions, encompassing social, professional, and community engagement barriers; and (4) Environmental and Personal Factors, which influence adaptation, including access to assistive devices and psychological support. This structured approach allows for a comprehensive evaluation of how rehabilitation interventions impact functional independence, participation, and quality of life. The outcomes from this spreadsheet were classified into an evidence map by outcome category. This evidence map displayed information regarding the outcomes of the Lewiston Rehabilitation Intervention. A descriptive and thematic analysis was conducted based on the reasons proposed by Arksey and O’Malley for conducting a scoping review. At each step, team members independently categorized the publications and then discussed the findings to reach a consensus.

## 11. Results

### 11.1. Selection of the Sources of Evidence

#### 11.1.1. Search Results

Our search encompassed four electronic databases—PubMed (*n* = 232), Web of Science (*n* = 38), Scopus (*n* = 78), and Google Scholar (*n* = 47)—yielding an initial pool of 395 records. After removing 67 duplicates, 328 records remained for title and abstract screening. This stage resulted in the exclusion of 165 articles that did not meet the inclusion criteria. Three additional eligible articles were identified from Google Scholar [9,10,11].

A total of 163 articles were sought for full-text retrieval, of which 73 could not be obtained. Ninety full-text articles were successfully retrieved and assessed for eligibility. During this stage, 77 articles were excluded for the following reasons:

(1) Wrong population, including pediatric or mixed-age samples (*n* = 18);
(2) Absence of a low-vision rehabilitation intervention (*n* = 22);
(3) Insufficient or non-extractable outcome data (*n* = 16);
(4) Manuscripts available only in non-English languages (*n* = 8);
(5) Inaccessible full text despite multiple retrieval attempts (*n* = 13).

**Figure 1:**
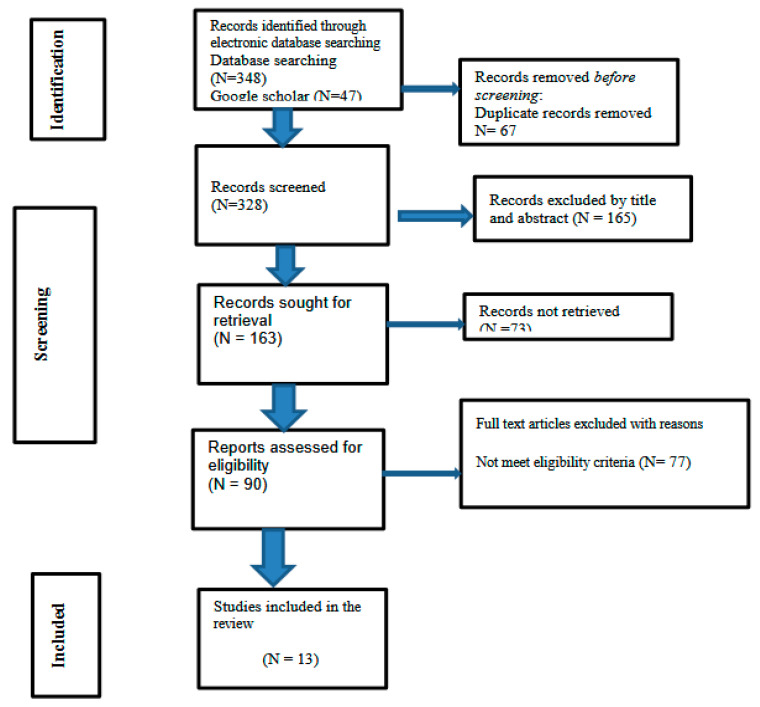
PRISMA flowchart describing the selection of studies mapping the existing literature on low-vision rehabilitation intervention.

These exclusion categories are detailed in the PRISMA 2020 [11] flow diagram (Figure 1).

Following this assessment, 13 primary studies met all eligibility criteria and were included in the final review. The review process was conducted in accordance with a predetermined protocol and reported following the Preferred Reporting Items for Systematic Reviews and Meta-Analyses extension for Scoping Reviews (PRISMA-ScR) guidelines (S1—PRISMA Checklist).

#### 11.1.2. Characteristics of the Included Studies

We identified 13 original, peer-reviewed articles and reviews published between 1 January 2003, and 25 December 2024 that focused on low-vision rehabilitation interventions, meeting our inclusion criteria. All the selected articles were published from 2004 onwards. Table 1 provides a complete list of the publications included in the review, including details such as author name, publication year, study area, study design, population, and sample size. Most of the articles describe randomized clinical trials (*n* = 10, 77%), and the included studies were conducted across multiple regions, including the USA, UK, the Netherlands, Australia, India, and Germany. The populations in the reviewed studies were primarily individuals older than 18 years.

## 12. Result of the Individual Study

The scoping review identified a range of studies on low-vision rehabilitation involving diverse populations and methodological designs. Randomized controlled trials (RCTs) were the predominant study type and were conducted in Australia [12,13], the United Kingdom [5,6], Germany [14,15], Pennsylvania (USA) [16], and other regions of the United States [17,18]. These studies targeted adults with low vision or specific clinical conditions such as age-related macular degeneration (AMD), with sample sizes ranging from 37 [14] to 192 [12] (Table 1).

Prospective cohort studies contributed significant insights, with primary studies in India [15], the USA [19], and Germany [20] examining populations ranging from 34 to 468 individuals. These studies addressed various visual impairments, including AMD and corneal diseases, emphasizing long-term functional outcomes (Table 1).

The populations studied in the scoping review ranged widely in terms of demographics and clinical criteria. Several studies focused on individuals with visual acuity worse than 20/100 but better than 20/500 [17,18,21]. Other studies targeted patients with homonymous visual field defects aged 18–75 years [22], while one study [13] concentrated on older adults (Table 1).

Sample sizes varied significantly, reflecting differences in study scope and design feasibility. Large-scale cohort studies, which included 468 participants [19], provided extensive data, whereas smaller RCTs [16], with 48 participants, and a different study [14] with 37 participants, offered detailed, focused findings. This diverse methodological and population framework underscores the multifaceted approach needed to address the complexities of low-vision rehabilitation (Table 1)

### 12.1. Synthesis of Outcomes

To improve interpretability across heterogeneous study designs, the outcomes of the included studies were synthesized according to the International Classification of Functioning, Disability, and Health (ICF) framework. This structure allowed for a coherent evaluation of the diverse intervention models and outcome measures reported across the review. For each ICF domain, we describe the outcomes assessed, the measurement instruments used, and the direction of the effects, with explicit alignment with the studies included in this review. The distribution of outcomes, corresponding measurement tools, and the key impacts across studies are summarized in Table 2, prior to the detailed domain-specific synthesis that follows.

**Description of Rehabilitation Interventions**

The 13 studies utilized diverse rehabilitation interventions, which were mapped in greater detail to improve clarity and comparability.

- Visual training interventions included structured reading exercises (e.g., MNREAD passages), eccentric viewing training, scanning training for visual field loss, and contrast-enhancement strategies.
- Magnification-based interventions involved optical magnifiers, electronic video magnifiers, and handheld or stand devices, with structured training sessions delivered by certified low-vision therapists.
- Multidisciplinary rehabilitation programs combined occupational therapy, psychological counseling, mobility training, and device training within a coordinated clinical model (e.g., Veterans Affairs low-vision programs).
- Home-based rehabilitation models included therapist-guided training delivered at participants’ homes, emphasizing ADL support, problem-solving strategies, and environmental modifications.
- Self-management programs focused on education, peer support, and coping strategies, delivered without intensive therapist involvement.

#### 12.1.1. Visual Function Outcomes

Visual function was the most frequently evaluated domain, with studies assessing visual acuity, contrast sensitivity, reading speed, visual field performance, and corneal clarity using standardized tools such as ETDRS and logMAR charts, the Pelli–Robson test, MNREAD and IReST passages, automated perimetry, and corneal grading scales. Across the evidence base, structured visual rehabilitation produced consistent improvements. Interventions combining magnification devices with therapist-led training enhanced reading speed in individuals with corneal disease ([9], while home-based programs for age-related macular degeneration improved reading fluency and contrast sensitivity and reduced depressive symptoms [20]. Low-vision device training in clinical settings increased reading endurance and functional acuity (Pearce et al. [21]), and visual field-specific training improved visual processing relevant to daily activities among patients with homonymous field defects [11]. Overall, randomized controlled trials such as those conducted by Acton et al. [5] and Reeves et al. [6] demonstrated the largest and most clinically meaningful gains, underscoring the effectiveness of structured, therapist-guided visual rehabilitation.

#### 12.1.2. Activity Limitations Outcome

Activity-related outcomes focused on performance in daily tasks, mobility, reading-related activities, and efficiency in completing functional goals. Across the reviewed studies, multidisciplinary rehabilitation programs produced consistent improvements in independence, task performance, and mobility, as shown in Gothwal and Bharani’s cohort from India [15]. Goal-directed visual training yielded substantial functional gains among patients with homonymous visual field defects, with Goal Attainment Scaling demonstrating meaningful improvements in activities of daily living (Elshout et al. [11]). High-intensity rehabilitation models used in Veterans Affairs programs showed large effect sizes across reading, mobility, and visual–motor integration (Stelmack et al. [15,19]). In contrast, self-management interventions produced minimal improvement in daily functional ability (Rees et al. [6]), highlighting the importance of therapist-led, structured rehabilitation for enhancing activity-level outcomes.

#### 12.1.3. Participation Restrictions Outcome

Participation-focused outcomes covering social engagement, emotional well-being, and vision-related quality of life showed favorable responses to several intervention models. Home-based rehabilitation in the UK led to improved vision-related quality of life and greater satisfaction with functional performance (Acton et al. [17]). Multidisciplinary rehabilitation produced notable improvements in emotional well-being, reduced frustration, and enhanced social participation in studies conducted in India (Gothwal & Bharani [21]) and the United States (Goldstein et al. [22]). Device-based interventions improved self-perceived task difficulty but showed no additional benefits with extended training sessions (Pearce et al. [10]). Self-management programs demonstrated little to no impact on participation or quality-of-life indicators, even at follow-up (Rees et al. [6]). In contrast, structured Veteran Affairs rehabilitation models showed durable improvements in quality of life that were sustained up to 12 months (Stelmack et al. [15,19]).

#### 12.1.4. Environmental and Personal Factors Outcome

Environmental and personal factors examined in the included studies encompassed device use, service satisfaction, emotional adaptation, and prevention of depressive symptoms. Home-based rehabilitation programs consistently achieved high satisfaction rates, especially when both functional and psychosocial needs were addressed (Acton et al. [17]). Interventions involving magnification devices resulted in measurable improvements in reading performance, although further device training did not yield additional benefit (Pearce et al. [10]). In AMD-specific interventions, home-based reading and visual training helped prevent depressive symptoms and supported emotional adaptation (Kaltenegger & Kuester [20]). Several studies, including those by Goldstein et al. [22] and Reeves et al. [18], also noted persistent barriers such as device fatigue, limited long-term use, and environmental factors such as inadequate lighting or accessibility challenges.

1. ADL = Activities of Daily Living; LVD = low vision device; VRQoL = vision-related quality of life; AMD = age-related macular degeneration.
2. Outcomes and measurement tools listed represent the most commonly reported assessments across included studies; individual studies may have used multiple tools.
3. Impacts summarize cross-study patterns rather than results from a single trial.

## 13. Research Gap, Outcomes, and Key Findings

The studies included in this scoping review evaluated diverse low-vision rehabilitation interventions and collectively demonstrated improvements across visual, functional, and psychosocial domains. Evidence from Australia [12] showed that low-vision rehabilitation enhanced daily living activities and overall quality of life, emphasizing the need to identify the most effective intervention components for specific visual impairment types. Complementary findings from India [15] further supported the benefits of multidisciplinary rehabilitation, with improvements noted in visual function, mobility, and emotional well-being (Table 3).

### 13.1. Identified Research Gaps

Several studies highlighted important gaps in the existing evidence base. Research from the Netherlands [22] indicated a lack of long-term follow-up data for visual training in individuals with homonymous visual field defects, despite demonstrating substantial short-term functional independence gains. Similarly, home-based rehabilitation interventions in the UK (Acton et al. [5]) showed promising improvements in visual function and user satisfaction, yet the sustainability and optimal configuration of such programs remain insufficiently explored.

### 13.2. Variability in Outcome Measures and Methodology

Findings from the United States underscored the need for standardized and universally accepted outcome measures. Goldstein et al. [19] reported that 44–50% of participants achieved clinically meaningful improvements in visual ability, demonstrating the value of validated measurement frameworks. Across studies, considerable variability in outcome measures, intervention types, and follow-up durations limited comparability and hindered the development of unified clinical recommendations.

### 13.3. Key Intervention-Specific Outcomes

Evidence from Germany demonstrated that home-based reading and visual training provided additional psychosocial benefits for individuals with age-related macular degeneration, including prevention of depressive symptoms (Kaltenegger & Kuester [14]). Further, device-based rehabilitation in patients with corneal diseases produced significant improvements in reading speed and performance (Oeverhaus et al. [20]). These findings demonstrate the potential of targeted interventions but also reveal the need for context-specific and population-specific research.

### 13.4. Integrated Evidence Interpretation

Across all ICF domains, the collective evidence indicates the following:

- Structured visual training, multidisciplinary rehabilitation, and home-based interventions consistently yield the most robust improvements in visual function, functional independence, and psychosocial well-being.
- Self-directed or low-intensity programs offer limited benefits, reinforcing the importance of therapist-guided, high-quality rehabilitation models.
- Outcome heterogeneity remains a substantial barrier, as studies frequently employ different measurement instruments for similar constructs, limiting cross-study comparability.
- Long-term outcomes are insufficiently examined, with most studies assessing short-term (1–6 months) effects and providing limited evidence on sustained rehabilitation gains.
- Psychosocial factors, including emotional well-being, adaptation, and social participation, remain underrepresented in both intervention design and assessment, despite their central role in coping with vision loss.

This synthesized interpretation highlights the need for standardized outcome measures, rigorous methodological designs, and expanded attention to psychosocial and long-term outcomes to advance the evidence base for low-vision rehabilitation practice.

## 14. Discussion

This scoping review synthesized evidence from a broad range of study designs, including exploratory, single-center, and multicenter randomized controlled trials (RCTs) [5,6,13,14,20], prospective cohort studies [15,19], and randomized crossover trials [22]. This methodological diversity reflects the growing rigor in evaluating low-vision rehabilitation, while also highlighting the challenges inherent in studying complex, individualized interventions.

Across studies, low-vision rehabilitation produced consistent improvements in visual function, activity performance, and quality of life (QoL). Structured visual training and magnification-based interventions improved contrast sensitivity, reading speed, and visual acuity, particularly for individuals with corneal disease [20] and age-related macular degeneration (AMD) [14]. Clinical device-based programs enhanced reading endurance and functional acuity [21], while visual field training led to measurable functional gains among individuals with homonymous hemianopia [22]. Multidisciplinary rehabilitation approaches, particularly those delivered in India [15] and in U.S. Veteran Affairs programs [17,18], yielded large improvements in mobility, ADLs, and visual–motor function. Home-based rehabilitation showed high satisfaction and functional benefit, with 44–50% of participants in U.S. studies achieving clinically meaningful improvements in visual ability [19].

Despite these positive outcomes, several research gaps persist. First, most studies included only short-term follow-up, limiting understanding of the durability of rehabilitation gains. Second, the wide variability in outcome measures such as VFQ-48, LVQOL, NEI-VFQ, VCM1, and the use of multiple reading assessments, hinders comparability and highlights the need for standardized tools. Third, psychosocial outcomes such as depression, emotional adjustment, and adaptation were inconsistently assessed, despite evidence of their importance in AMD populations [14] and multidisciplinary programs [15]. Fourth, population diversity was limited; few studies included underrepresented groups or individuals in low-resource settings. Fifth, intervention fidelity, intensity, and delivery characteristics were rarely reported, restricting the interpretation of dose–response relationships. Finally, self-management programs showed minimal benefit in QoL outcomes compared with therapist-led interventions [13].

Overall, the findings reinforce that high-intensity, therapist-guided rehabilitation provides the most consistent benefits across the visual, functional, and psychosocial domains. Future research should prioritize long-term follow-up, standardized outcome frameworks, comprehensive psychosocial assessment, and inclusion of socioeconomically and culturally diverse populations. Greater attention to intervention fidelity and implementation barriers is also essential for advancing evidence-based, equitable low-vision rehabilitation.

## 15. Conclusions

This review predominantly focuses on randomized controlled trials (RCTs). The interventions examined included visual training and multidisciplinary approaches that combined different therapeutic modalities. This scoping review demonstrates that low-vision rehabilitation, particularly structured visual training, magnification-based interventions, multidisciplinary programs, and home-based models, consistently improves visual acuity, reading speed, contrast sensitivity, functional independence, and vision-related quality of life. Evidence from randomized trials and prospective studies indicates that a substantial proportion of adults with low vision achieve clinically meaningful functional gains when rehabilitation is therapist-guided and delivered with sufficient intensity. However, the evidence base remains limited by short follow-up periods, heterogeneous outcome measures, inconsistent assessment of psychosocial factors, and underrepresentation of diverse populations. Self-management interventions showed minimal benefit, reinforcing the need for professionally guided approaches.

In conclusion, there is a clear need for standardized evaluation frameworks, longer-term studies, and more inclusive research designs. Strengthening methodological consistency and addressing psychosocial and contextual factors will be essential to improving the accessibility, equity, and effectiveness of low-vision rehabilitation.

## 16. Limitation

Limitations of this review include the restriction to English-language studies and the possibility that relevant articles were missed despite manual searching. Nonetheless, the review provides a coherent synthesis of the current evidence and identifies priority research gaps for improving low-vision rehabilitation.

## Figures and Tables

**Table 1:** Summary of the study design, population, and sample size for the outcomes of low-vision rehabilitation.

Author Name	Publication Year	Study Area	Study Design	Population	Sample Size
Lamoureux EL et al.	2007	Australia	Randomized controlled trial	Individuals with low vision, age 18 years and above.	192
Elshout JA et al.	2018	Netherlands	Prospective cohort study	Patients with homonymous visual field defects between 18 and 75 years of age.	40
Gothwal VK, Bharani S	2015	India	Prospective cohort study	Adults with low vision.	255
Acton JH et al.	2016	United Kingdom	Exploratory randomized controlled trial	Adults with low vision.	67
Goldstein JE et al.	2015	USA	Prospective cohort study	Adults with low vision treated at outpatient clinical centers.	468
Kaltenegger et al.,	2019	Germany	Randomized controlled trial	Patients with age-related macular degeneration (AMD).	37
Oeverhaus M et al.,	2020	Germany	Prospective, randomized cross-over trial	Patients with visual impairment due to corneal diseases.	34
Rees G et al.	2015	Australia	Randomized controlled trial	Older adults.	153
Draper EM et al.	2016	Pennsylvania	Randomized controlled trial	40 years of age and older.	48
Reeves BC et al.	2004	UK	A single-center parallel group randomized controlled trial	Individuals referred for low-vision rehabilitation with a primary diagnosis of age-related macular degeneration (AMD), visual acuity worse than 6/18 in both eyes, and equal to or better than 1/60 in the better eye.	194
Stelmack JA et al.	2008	USA	Multicenter randomized clinical trial	Visual acuity in the better-seeing eye worse than 20/100 and better than 20/500.	126
Stelmack JA et al.	2012	USA	Prospective study (RCT)	Visual acuity in the better-seeing eye worse than 20/100 and better than 20/500 and were eligible for Veterans Affairs (VA) services.	100
Pearce E et al.	2011	UK	Randomized controlled trial	Visual acuity in the better-seeing eye worse than 20/100 and better than 20/500 and were eligible for Veterans Affairs (VA) services.	96

**Table 2:** Summary of outcomes, measures, and impacts across ICF domains.

ICF Domain	Outcomes Assessed	Measurement Tools	Studies Impact
Visual Function	Visual acuity Contrast sensitivity Reading speed/accuracy Visual field function Corneal clarity	ETDRS/logMAR charts Pelli–Robson MNREAD/IReST Automated perimetry Corneal grading scales	Improved reading speed and acuity Enhanced contrast sensitivity Better visual processing in daily tasks
Activity Limitations	ADLs Mobility Task efficiency Reading-related tasks	VFQ-48 ADL assessments VA VFQ-48 Task performance tests GAS	Increased independence and mobility Improved task performance Functional gains in hemianopia and veteran populations
Participation Restrictions	VRQoL Social participation Emotional well-being Functional self-perception	LVQOL NEI-VFQ VCM1 SF-36 Emotional well-being scales	Enhanced quality of life Improved emotional and social participation Limited impact of self-management programs
Environmental and Personal Factors	LVD use/adoption Service satisfaction Adaptation to vision loss Depression prevention	LVD usage logs Satisfaction surveys Adaptation scales Psychological screening tools	High satisfaction with home-based care Better reading outcomes with LVDs Prevention of depressive symptoms in AMD

**Table 3:** Summary of research gaps, outcomes, and key findings for the outcomes of low-vision rehabilitation.

Author Name	Publication Year	Study Area	Research Gaps	Outcomes	Key Finding
Lamoureux EL et al.	2007	Australia	Need for understanding specific components of rehabilitation for different types of vision impairment.	Participation in daily living activities and quality of life (QoL) improvement.	Low-vision rehabilitation improved participation in daily living activities and QoL. Participants in the intervention group showed greater improvements compared to the control group.
Elshout JA et al.	2018	Netherlands	Lack of studies examining long-term effects of visual training on ADLs and QoL in patients with homonymous visual field defects.	Improvement in activities of daily living (ADLs).	Visual training significantly improved activities of daily living as measured by Goal Attainment Scaling (GAS). The intervention led to a positive impact on functional independence.
Gothwal VK, Bharani S	2015	India	Limited data on the effectiveness of multidisciplinary approaches for low-vision rehabilitation in diverse populations.	Visual function improvement, quality of life (QoL) improvement, and participation in daily living activities.	Multidisciplinary low-vision rehabilitation improved visual function, QoL, and participation in daily living activities. Significant improvements in mobility, independence, and emotional well-being.
Acton JH et al.	2016	United Kingdom	Limited research on the effectiveness of home visit- based low-vision interventions for improving visual function.	Visual function outcomes (e.g., contrast sensitivity, visual acuity) and participant satisfaction.	Home visit-based low-vision rehabilitation showed significant improvements in visual function out- comes (contrast sensitivity, visual acuity). Participants reported high satisfaction with the intervention.
Goldstein JE et al.	2015	USA	Need for standardized measures of clinically meaningful rehabilitation outcomes for low-vision patients.	Visual ability and in visual ability in four functional domains as measured by the Activity Inventory.	44–50% showed clinically meaningful differences in overall visual ability after low-vision rehabilitation (LVR), and the average effect sizes in overall visual ability were large, close to 1 SD.
Kaltenegger et al.,	2019	Germany	Limited understanding of how home-based interventions can improve quality of life and functional outcomes in AMD patients; need for larger sample sizes and long-term follow-up studies to validate findings.	Reading ability and quality of life.	Patients with AMD who already use magnifying aids benefit from additional RT and it can contribute to preventing depression and improve QoL.
Oeverhaus M et al.,	2020	Germany	Limited data on visual rehabilitation specifically for patients with corneal diseases; need for long-term evaluation of LVA effectiveness in this population.	Reading speed assessed with International Reading Speed Texts (IReST); patient-reported ratings of LVAs; correlation between corneal haze and visual acuity.	LVAs significantly improved reading speed in patients with corneal diseases.
Rees G et al.	2015	Australia	Limited data on the long-term effectiveness and scalability of self-management programs in this population.	Limited benefit of an LVSM program on QoL for older adults.	At one- and six-month follow-up assessments, no significant between-group differences were found for vision-specific QoL, emotional well-being, adaptation to vision loss or self-efficacy (*p* > 0.05). Univariate and multivariate analyses revealed no impact of the intervention on outcome measures.
Draper EM et al.	2016	Pennsylvania	Not explicitly stated; however, the study suggests a need for further research into the psychosocial factors influencing rehabilitation outcomes and the effectiveness of different rehabilitation settings.	Primary outcome: change in functional vision in activities of daily living, assessed with the Veteran’s Administration Low-Vision Visual Function Questionnaire (VFQ-48), including overall visual ability and specific domains (reading, mobility, visual information processing, and visual motor skills).	Both clinic-based and home-based groups showed significant improvement in overall visual ability at the final visit compared with baseline.
Reeves BC et al.	2004	UK	The study highlights the need for preliminary evidence of effectiveness before proposing new low-vision rehabilitation interventions, given the lack of effectiveness observed in the enhanced model evaluated.	Primary outcome: Vision-specific quality of life (QoL) measured byVCM1. Secondary outcomes: generic health-related QoL (SF-36), psychological adjustment to vision loss, measured task performance, restriction in everyday activities, and use of low-vision aids (LVAs).	Except for generic health-related QoL (SF-36) and physical and mental component summary scores, outcomes did not differ significantly for any of the outcomes. Differences in vision-specific quality of life (QoL (VCM1) were ELVR (enhanced low-vision rehabilitation model) vs. CLVR (conventional low-vision rehabilitation), 0.06 (95% CI to 0.17 to 0.30, *p* = 0.60); ELVR vs. CELVR, 0.12 (95% CI to 0.11 to 0.34, *p* = 0.31); CELVR (CLVR with home visits that did not include rehabilitation) vs. CLVR, −0.05 (95% CI −0.29 to 0.18, *p* = 0.64). Differences in the SF-36 favored CLVR compared to ELVR (ELVR vs. CLVR: physical = −6.05, 95% CI −10.2 to− 1.91, *p* = 0.004; mental = −4.04, 95% CI −7.44 to −0.65, *p* = 0.02). At 12 months, 94% of participants reported using at least one LVA.
Stelmack JA et al.	2008	USA	The effectiveness of such programs in a veteran population, in which prior studies were limited.	The primary outcome was vision-related quality of life, measured through questionnaires, and secondary outcomes included visual functioning (acuity, contrast sensitivity) and mobility.	The treatment group demonstrated significant improvement in all aspects of visual function compared with the control group.

## Data Availability

The data presented in this study are derived from previously published articles, all of which are cited in the reference list. No new datasets were generated or analyzed.

## Supplementary Materials

The following supporting information can be downloaded at: https://www.mdpi.com/article/10.3390/vision10010003/s1, [23].
